# Electronic and Optical Properties of 2D-TMD/Janus Heterostructures Under the Influence of an Electric Field: First-Principles Calculations

**DOI:** 10.3390/ma18235378

**Published:** 2025-11-28

**Authors:** Daulet Sergeyev, Ainur Duisenova, Kuanyshbek Shunkeyev

**Affiliations:** 1 Department of Physics, K.Zhubanov Aktobe Regional University, Aktobe 030000, Kazakhstan; 2 Department of General Engineering Disciplines, T.Begeldinov Aktobe Aviation Institute, Aktobe 030012, Kazakhstan

**Keywords:** DFT, Janus heterostructures, van der Waals interactions, MoS_2_/SeMoS, MoSe_2_/SMoSe, dielectric permittivity, electric field, optical properties

## Abstract

This work presents the results of a theoretical investigation of the electronic and optical properties of van der Waals Janus nanoheterostructures MoS_2_/SeMoS and MoSe_2_/SMoSe, carried out within the framework of density functional theory (DFT) using the generalized gradient approximation (GGA-PBE) together with the Grimme-D3 dispersion correction. The calculated band structures show that both heterostructures possess an indirect bandgap whose magnitude is highly sensitive to an external electric field. In the MoS_2_–SeMoS system, increasing the applied field leads to a gradual narrowing of the bandgap and a transition to a metallic state at approximately 75 V, whereas in MoSe_2_–SMoSe, the bandgap first increases (up to 20 V) and then decreases, indicating a nonlinear field-dependent behavior. Analysis of the dielectric function reveals an enhancement of the static dielectric permittivity and a red shift in the absorption spectra with increasing field strength, which can be attributed to charge redistribution and an increased contribution from ionic polarizability. These results demonstrate the possibility of effectively controlling the bandgap width, polarizability, and optical response of Janus nanoheterostructures using an external electric field. This opens up promising prospects for their application in tunable photodetectors, light modulators, valleytronic components, and next-generation optoelectronic systems.

## 1. Introduction

Over the past decades, there has been substantial interest in the study of low-dimensional structures, as their unique properties open up broad prospects for applications in nanoelectronics, optoelectronics, and quantum technologies [1,2,3]. Such structures include zero-dimensional systems, in which the motion of charge carriers is confined in all three spatial directions [4,5,6]; one-dimensional systems—nanowires and nanotubes—characterized by confinement in two directions [7,8,9]; and two-dimensional systems, such as thin films and graphene-like materials, where carrier motion is restricted to a single plane [10,11,12]. Investigating the properties of these systems enables a deeper understanding of quantum effects at the nanoscale and supports the development of new functional materials with tailored characteristics.

Two-dimensional materials, which represent atomically thin layers in which the motion of charge carriers is confined in only one spatial direction, are of particular interest among low-dimensional systems. Owing to their high carrier mobility, exceptional mechanical strength and flexibility, and the ability to precisely tune their electronic properties, two-dimensional materials offer new opportunities for the development of miniaturized and highly efficient electronic and optoelectronic devices. Particular attention has been given to two-dimensional semiconducting materials such as transition metal dichalcogenides (TMDs)—for example, MoS_2_, WS_2_, MoSe_2_, and related compounds—which exhibit a direct bandgap in the monolayer limit and pronounced quantum effects. The low-temperature properties of certain TMD materials, including NbS_2_, NbSe_2_, TaS_2_, and TaSe_2_, have also attracted considerable interest, as they display superconducting behavior below their critical temperatures [13,14]. The observed superconductivity in these systems provides a platform for exploring the fundamental mechanisms of the superconducting state in low-dimensional (1D and 2D) structures. Such systems enable the study of unique quantum phenomena arising from reduced dimensionality, as well as the interactions among electrons, phonons, and spin degrees of freedom under strong correlation conditions [15,16,17]. These materials combine the advantages of two-dimensional systems with the ability to tailor their electronic structure, making them promising candidates for next-generation transistors, photodetectors, and other nanoelectronic devices [18,19,20].

A special place among two-dimensional semiconducting materials is occupied by van der Waals (vdW) TMDs, whose atomic layers are held together by weak vdW interactions. Such a structure eliminates dangling bonds on the surface and enables the facile formation of heterostructures through the layer-by-layer assembly of different two-dimensional crystals. This makes it possible to create materials with predetermined properties that cannot be realized in conventional three-dimensional crystals. As a result, it becomes feasible to design “artificial crystals” with tailored electronic and optical characteristics, including controlled band alignment, the generation of interlayer excitons, and the manipulation of charge transfer [21,22].

Of particular interest in this field are heterostructures composed of 2D TMD layers and the recently synthesized Janus TMD structures (e.g., MoSSe, WSSe) [23,24,25]. Janus structures possess intrinsic asymmetry, as their two surfaces are terminated by different chalcogen atoms, giving rise to an internal dipole moment and an asymmetric potential distribution. When such structures are combined with conventional 2D TMD layers, TMD/Janus nanoheterostructures are formed.

At present, TMD/Janus nanoheterostructures are highly relevant for the development of advanced nanoelectronic and optoelectronic devices. Their intrinsic asymmetry, combined with interlayer interactions, can lead to tunable band structures and optical transitions through strong coupling and energy shifts, as well as to the formation of unique types of excitons with spatially separated charge carriers (interlayer excitons) and a local electric field at the interface, which can be exploited for the efficient separation of photogenerated electron–hole pairs. However, despite their strong potential, the influence of an external electric field on the electronic and optical properties of nanoheterostructures composed of a 2D TMD layer and a Janus structure remains insufficiently explored and requires detailed investigation. The application of an external electric field represents one of the most effective and non-invasive methods for controlling and dynamically tuning the properties of 2D materials. In the case of TMD/Janus nanoheterostructures, the external field can modulate the magnitude and direction of the intrinsic dipole moment of the Janus layer, finely tune the potential barrier and the overlap of wave functions between the layers, and modify the lifetime and energy of excitons—all of which are critically important for photodetectors and light-emitting diodes.

It should be noted that the required properties of modern nanoscale devices can be tuned through various parameters such as an external electric field [26,27,28], the twist angle between the layers [29,30,31], the intrinsic dipole moment [32,33], mechanical strain [34,35,36], and others. For one-dimensional vdW nanostructures (e.g., MoS_2_(n,n)@MoSe_2_(n,n)), the electronic and optical characteristics can be finely adjusted by selecting an appropriate chirality index [37]. In nanostructures based on the combination of 2D TMDs with Janus interfaces (e.g., MoS_2_/SeMoS), it becomes possible to control several independent parameters simultaneously. This opens pathways for the creation of tunable interlayer-exciton light sources, modulators, and photodetectors with dynamically reconfigurable characteristics, as well as elements of valleytronics [38,39] and excitonic logic [40]. Recent studies demonstrate the feasibility of electrically controlling emission directionality and polarization, observing significant Stark shifts, and enhancing exciton lifetimes, including the integration of such systems with optical microresonators [41].

Given the complexity and quantum nature of phenomena at the atomic scale, experimental data must be complemented and supported by theoretical studies. To achieve a deep understanding of the mechanisms governing the interaction of an external electric field with the electronic structure and dipole moments in these heterostructures, it is essential to employ first-principles (ab initio) methods based on DFT [42,43,44]. Such an approach makes it possible to accurately compute field-induced band-structure shifts, variations in the effective masses of charge carriers, and modifications of optical transitions, which represent key steps toward the practical implementation of these systems in applications such as logic elements, gate-tunable nanotransistors, and high-efficiency photovoltaic devices.

In this work, the electronic and optical properties of vdW quasi-two-dimensional heterostructures composed of a transition metal dichalcogenide monolayer and a Janus structure (MoS_2_/SeMoS and MoSe_2_/SMoSe) are investigated within the framework of DFT. The influence of an external electric field on their band structures and optical absorption spectra is systematically analyzed.

## 2. Structural Model and Geometry

**Figure 1:**
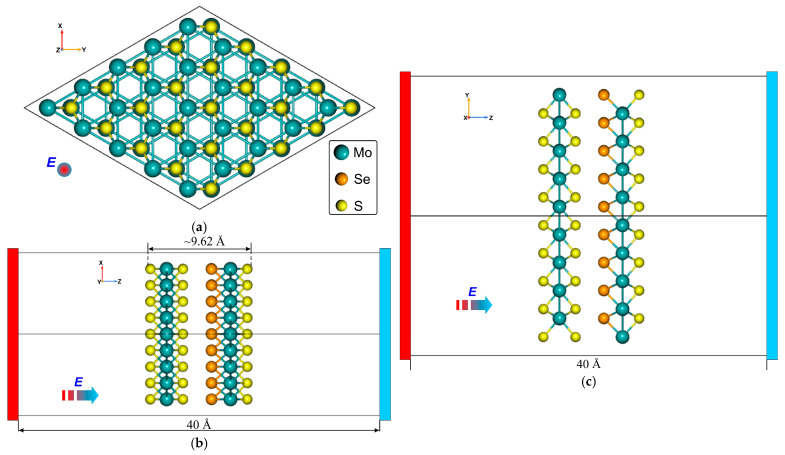
Geometry of the nanoheterostructure MoS_2_/SeMoS: (**a**) XY-plane; (**b**) ZX-plane; (**c**) ZY-plane. (E—applied external electric field).

**Figure 2:**
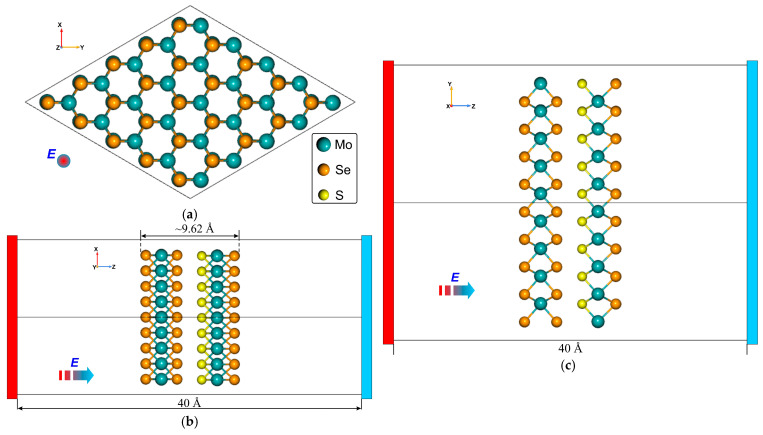
Geometry of the nanoheterostructure MoSe_2_/SMoSe: (**a**) XY-plane; (**b**) ZX-plane; (**c**) ZY-plane. (E—applied external electric field).

The geometries of the optimized vdW Janus nanoheterostructures MoS_2_/SeMoS and MoSe_2_/SMoSe are shown in Figure 1 and Figure 2, respectively. Among the various Janus configurations examined, these two systems were selected because the sulfur and selenium atoms occupy closely aligned positions, giving rise to a stable intrinsic dipole moment. Such an atomic arrangement results in pronounced layer polarization and enhances the sensitivity of the structures to an external electric field. The interlayer distance between the TMD sheets in the MoS_2_/SeMoS and MoSe_2_/SMoSenanoheterostructures ranges from 2.9 to 3.2 Å. It is known that among all possible stacking configurations of TMD layers, the C7-type stacking (see, for example, Ref. [45]) provides the highest energetic stability. In this configuration, the chalcogen atoms of one monolayer are positioned directly above the transition-metal atoms of the adjacent monolayer, as illustrated in Figure 1 and Figure 2. Therefore, in what follows, we focus exclusively on these energetically favorable stacking geometries.

The geometry optimization of the nanostructures and the description of interatomic interactions were performed within the framework of DFT, using the generalized gradient approximation GGA-PBE as the exchange–correlation functional [46]. The structures were optimized with force and stress convergence thresholds of 0.02 eV/Å and 0.001 eV/Å^3^, respectively, and a plane-wave energy cutoff of 75 Hartree.

It should be noted that in the present work the geometric structures of all TMD/Janus heterostructures were fully optimized in the absence of an external electric field. Once the minimum-energy configuration was obtained, subsequent calculations of the electronic and optical properties were carried out by applying a uniform perpendicular electric field of varying magnitude to the fixed atomic geometry.

This approach is widely adopted in studies of two-dimensional materials when the objective is to examine the electronic response of the system under a preserved interlayer configuration rather than to model a full structural rearrangement induced by the field. A similar computational protocol has been employed in numerous studies of TMD monolayers and heterostructures. Liu et al.demonstrated that the geometry of bilayer MoS_2_ can be optimized in the absence of an external electric field (at E = 0) and subsequently used for field-dependent calculations because geometric distortions exert a negligibly small influence on the electronic structure [47]. The same methodology is used for modeling GaS monolayers under a perpendicular electric field [48], TMD bilayers in the context of the giant Stark effect [49], and Janus-based heterostructures [50]. Thus, applying an external electric field to a fixed optimized geometry correctly captures the physical mechanism governing field-induced modifications of the band structure and optical response within the electronic-reaction regime, and reliably reveals the trends associated with the field perturbation.

## 3. Methods

In this work, the optical properties of the optimized vdWnanoheterostructures based on TMDs were calculated within the framework of DFT-GGA. The exchange–correlation interactions were treated with the Perdew–Burke–Ernzerhof (PBE) functional [51]. Although this functional is known to systematically underestimate the band gapsof semiconductors, its use is justified for the purposes of this study: our focus is on the relative changes in the electronic and optical properties under an external electric field rather than on the accurate reproduction of absolute bandgap values. According to literature reports on MoS_2_, WS_2_, and their Janus analogues, the 2H phase exhibits broad and delocalized d-states of the transition metal, indicating weak electronic correlations [49,50]. This suggests that the application of GGA-PBE is appropriate and sufficient for the systems considered here.

For the dielectric function and related optical characteristics, the Kubo–Greenwood equation was employed, which enables the calculation of the frequency-dependent dielectric susceptibility of the system [52,53]:

(1) $$\chi_{ij}\left(\omega \right)=-\frac{e^{2}\hbar^{4}}{m_{e}^{2}\varepsilon_{0}V\omega^{2}}\sum_{nm}\frac{f\left(E_{m}\right)-f\left(E_{n}\right)}{E_{nm}-\hbar \omega -i\hbar \Gamma }\pi_{nm}^{i}\pi_{mn}^{j}$$

where $\pi_{nm}^{i}$ is i component of the dipole matrix element between states n and m, V the volume, Γ the broadening, and $f\left(E\right)$ is the Fermi distribution function of quasiparticle energy, e is the charge of the electron, ℏ is Planck’s constant, E is energy, $\varepsilon_{0}$ is dielectric constant of vacuum, ω is frequency, $m_{e}$ is mass of the electron.

On the basis of Equation (1), the frequency (or energy) dependence of the dielectric permittivity was obtained, which reflects the optical response of the system under study:

(2) $$\varepsilon \left(\omega \right)=1+\chi \left(\omega \right),$$

The real part of the dielectric function characterizes the dispersion properties of the material and determines the phase shift in the transmitted electromagnetic radiation, whereas the imaginary part is associated with light absorption and describes the energy losses within the system.

The extinction coefficient k and the refractive index n of the nanoheterostructures were determined using the following expressions:

(3) kω=Re(ε)2+Im(ε)2−Re(ε)2,

(4) nω=Re(ε)2+Im(ε)2+Re(ε)2.

Based on expression (3), the optical absorption coefficient α(ω) was calculated, which characterizes the attenuation of the electromagnetic wave as it propagates through the nanoheterostructures under study.

(5) $$\alpha =2\frac{\omega }{c}k.$$

Analysis of the obtained dependencies makes it possible to identify the features of electronic transitions between the valence and conduction bands, as well as to determine the energy ranges in which the most intense absorption occurs. These findings are essential for understanding the mechanisms of light absorption and for evaluating the potential of the studied vdWnanoheterostructures in optoelectronic applications.

In this work, the Grimme-D3 method (dispersion correction with three-body terms) was employed to accurately describe dispersion interactions. This approach is based on adding an empirical dispersion correction to the DFT total energy. The correction accounts for long-range correlation effects (van der Waals forces) through a sum of pairwise atom–atom interactions with damping functions, as well as the three-center Axilrod–Teller–Muto terms. Compared to earlier versions, such as Grimme-D2, the Grimme-D3 scheme offers a more universal parameterization, providing improved predictive accuracy for a wide range of molecular and solid-state systems [54].

The band structure of the vdW nanoheterostructure based on a TMD was calculated using the OMX package (version 2015.1). The calculations were performed along the high-symmetry Γ–M–K–Γ path of the first Brillouin zone. A strict convergence criterion was applied for the SCF procedure, with the total energy required to converge to 10^−7^ eV. A multi-grid solver was employed to solve the Poisson equation, using Dirichlet boundary conditions along the C axis and periodic boundary conditions in the remaining directions.

In the calculation of the optical characteristics, the expansion of the Kohn–Sham orbitals was performed using the OMX basis set. The electronic wave functions were expanded with a lattice cutoff energy of 75 Hartree. For the Brillouin zone integration, a 15 × 15 × 1 k-point mesh was employed, generated according to the Monkhorst–Pack scheme [55].

The real and imaginary parts of the dielectric permittivity function were calculated using a 30 × 30 × 1 optical k-grid. A Gaussian broadening of 0.1 eV was applied to smooth the spectra. The calculations were performed in the energy range from 0 to 5 eV, as this interval is most relevant for photovoltaic and optoelectronic applications. The principal interband transitions that determine the absorption and reflection spectra in the visible and near-ultraviolet regions are located within this energy window. Analyzing the optical response in this range enables the evaluation of light-to-electricity conversion efficiency and the assessment of the potential suitability of the studied vdWnanoheterostructures for photovoltaic device applications.

The electronic and optical characteristics of the investigated nanoheterostructures were modeled using the AtomistixToolKit with Virtual NanoLab software package (version 2015.1) [56]. All simulations were carried out on a Supermicro server (Super Micro Computer, Inc., San Jose, CA, USA, distributor PolyWorks Company) equipped with an Intel^®^Xeon^®^Silver processor, 512 GB of RAM, and 64 cores.

## 4. Results and Discussion

**Figure 3:**
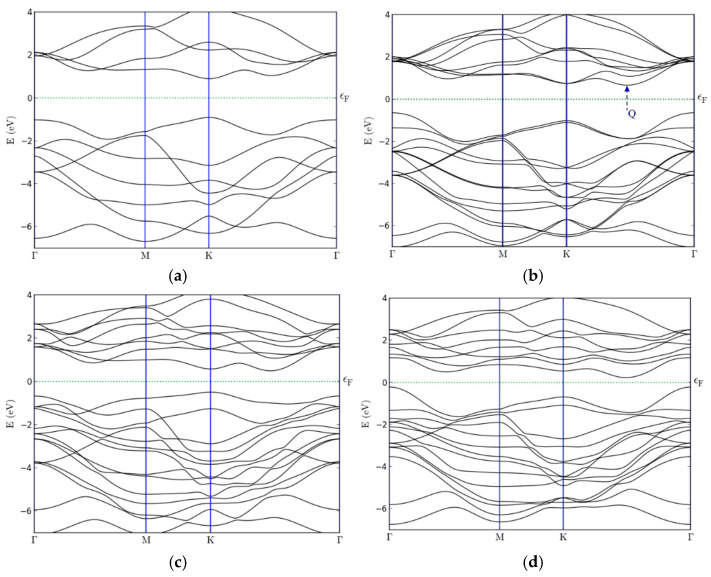
Band structure of the TMD/Janus nanoheterostructures: (**a**) 1L-MoS_2_; (**b**) 2L-MoS_2_; (**c**) MoS_2_-SeMoS; (**d**) MoSe_2_-SMoSe.

We now analyze the results of the calculated band structures of the TMD nanostructures (Figure 3a–d). For comparison, Figure 3a,b present the band structures of single-layer (1L-MoS_2_) and bilayer (2L-MoS_2_) molybdenite, computed using the methods described in Section 3. Analysis of the obtained energy spectra makes it possible to trace the influence of interlayer interactions and heterostructure formation on the positions of the valence and conduction bands, as well as on the characteristics of the bandgap.

It is well known that single-layer TMD nanostructures exhibit a direct bandgap [57]. For example, in 1L-MoS_2_ the energy difference between the valence-band maximum (VBM) and the conduction-band minimum (CBM) is approximately 1.8 eV and corresponds to the K–K transition (Figure 3a). Upon the addition of a second MoS_2_ layer, a lowering of the CB edge occurs at the Q point (Figure 3b). This feature, indicated in the figure by the point Q, is consistent with the results reported in Ref. [58]. As a consequence, the bandgap becomes indirect (Γ–K transition), and its magnitude decreases to about 1.3 eV.

It should be noted that when the vdW correction of Grimme-D3 is taken into account, the CB droop at the Q point becomes more pronounced, suggesting that interlayer (vdW) interactions contribute to the emergence of the indirect-gap character. A similar behavior is observed for mixed vdW nanoheterostructures composed of conventional and Janus monolayers (Figure 3c,d). For instance, in the Janus nanoheterostructure MoS_2_/SeMoS, the upper part of the valence band lies near the Q point (at approximately −0.5 to −1.0 eV), while the CB minimum is located near the K point, slightly above the Fermi level (≈0.5–1.0 eV). This indicates an indirect bandgap of the K–Q type with a magnitude of approximately 0.97 eV (Figure 3c). The band structure of the other Janus nanoheterostructure, MoSe_2_–SMoSe, differs somewhat: the VB maximum is located near the Γ point, whereas the CB minimum lies in the region of the Q point, resulting in a reduced bandgap of 0.425 eV (Figure 3d).

For the MoS_2_/SeMoS nanostructure, a local maximum of the valence band and a local minimum of the conduction band are observed near the K point, with a bandgap of approximately 1.06 eV. This suggests the possibility of band inversion or a transition to a direct-gap state upon the application of an external electric field.

**Figure 4:**
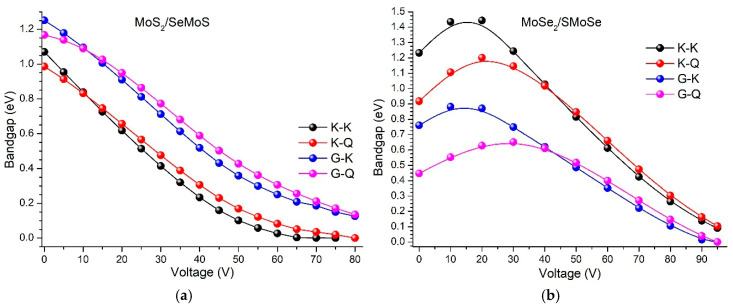
Modulation of the bandgap width under the influence of bias voltage: (**a**) MoS_2_/SeMoS; (**b**) MoSe_2_/SMoSe.

We now analyze the evolution of the bandgap width of the MoS_2_/SeMoS nanostructure under an applied external electric field, as well as the changes in the type of transitions between different points of the Brillouin zone (K–K, K–Q, Γ–K, Γ–Q) (Figure 4a).

As can be seen from the presented data, the MoS_2_/SeMoS structure exhibits a monotonic decrease in the bandgap width for all considered transitions as the applied voltage increases. Such behavior indicates the sensitivity of the electronic structure to the external field and demonstrates the possibility of controllably tuning the transition type (from indirect to direct) through electrostatic modulation. In the voltage range of 0–50 V, the bandgap decreases almost linearly. With a further increase in the applied field above 50 V, the narrowing process slows down and gradually approaches saturation, while at approximately 75 V the bandgap collapses, corresponding to a transition of the system into a metallic or semimetallic state (Figure 4a). It is noteworthy that even at voltages above 10 V, the indirect K–Q transition transforms into a direct K–K transition, indicating the feasibility of an electrically controlled semiconductor–metal transition.

Next, we examine the evolution of interband transitions between different points of the Brillouin zone as the external electric field is increased. As shown in Figure 4a, the K–K transition exhibits the lowest bandgap energy at voltages above 10 V and the fastest gap closure with increasing field (black curve). For the K–Q transition, the energy is slightly higher than that of K–K, yet the behavior is similar—the bandgap decreases rapidly and approaches nearly zero at approximately 80 V (red curve). The Γ–K transition is characterized by a wider bandgap (~1.2 eV) at zero voltage and shows a gradual decrease as the field increases, while still remaining larger than those of the K–K and K–Q transitions over the same voltage range (blue curve). The largest bandgap energy among all considered transitions (~1.1–1.2 eV at 0 V) is observed for the Γ–Q transition (pink curve); although it also decreases with increasing voltage, it remains the largest up to roughly ≈80V.

Thus, at low voltages (0–10 V), the heterostructure retains its semiconducting character with an indirect interband transition (K–Q). At intermediate voltages (10–60 V), a gradual reduction in the bandgap width is observed, accompanied by a transformation of the indirect gap into a direct one (K–K), which may lead to noticeable changes in the optical and electronic properties of the system. With a further increase in voltage—up to 80 V—the bandgap almost completely collapses, indicating a possible transition into a metallic state.

The K–K transition retains the lowest energy among all the transitions considered throughout the entire calculation range, which allows it to be regarded as the fundamental bandgap of the MoS_2_–SeMoS structure.

Thus, the evolution of the bandgap width in the MoS_2_/SeMoS heterostructure under an external electric field demonstrates a pronounced effect of electric-field control over the band structure, making such vdW systems promising for tuning optoelectronic properties and for the development of functional nanoscale devices with controllable characteristics.

We now analyze the evolution of the bandgap width in the MoSe_2_/SMoSe nanostructure under an external electric field, as well as the nature of the transitions between different points of the Brillouin zone (K–K, K–Q, Γ–K, Γ–Q) (Figure 4b). In contrast to the previous case (MoS_2_/SeMoS), a nonlinear behavior of the bandgap width is observed here. At low voltages (0–15 V), the bandgap initially increases, reaching a maximum at approximately 20 V, after which a gradual decrease is observed as the voltage continues to rise. At voltages above ~90–95 V, all the curves approach zero, indicating a transition of the system into a metallic state. It is worth noting that at voltages above 40 V, the transition type changes—from Γ–Q to Γ–K—which points to a modification of the interband transitions under the influence of the external electric field and confirms the high sensitivity of the MoSe_2_/SMoSe electronic structure to electrostatic perturbation.

Let us examine the evolution of interband transitions between different points of the Brillouin zone as the external electric field is increased (Figure 4b). At zero voltage, the K–K transition exhibits the largest bandgap width (~1.2 eV), which increases to ~1.4 eV at around 15 V, after which it gradually decreases and eventually closes at approximately 95 V (black curve). For the K–Q transition, the bandgap is ~0.9 eV at 0 V, increases to ~1.2 eV at ~20 V, and then begins to decrease. In the voltage range of 40–80 V, the values for the K–K and K–Q transitions become comparable, indicating their competition in defining the band-edge states (red curve).

The Γ–K transition starts with a bandgap width of about 0.8 eV, reaches a maximum of ~0.9 eV at around 15 V, and subsequently decreases linearly with increasing voltage (blue curve). The fundamental bandgap at low voltages corresponds to the Γ–Q transition (violet curve); however, at voltages above 40 V, a transition occurs, and the Γ–K path becomes dominant. Thus, in the MoSe_2_/SMoSe nanostructure, the fundamental bandgap is indirect (Γ–Q) at low and intermediate voltages (0–40 V), while at voltages above 40 V, it is formed by the Γ–K transition. At higher voltages (70–90 V), the bandgap closes, marking the transition of the material into a metallic state. It is worth noting that similar behavior is characteristic of the one-dimensional vdW analogues of the studied nanoheterostructures—coaxially connected nanotubes WS_2_(6,6)@MoS_2_(14,14) and WS_2_(8,8)@MoS_2_(16,16) [59]. As the external electric-field voltage increases, the bandgap width gradually decreases, leading to a semiconductor–metal transition at voltages of approximately 16 V and 18 V, respectively.

**Figure 5:**
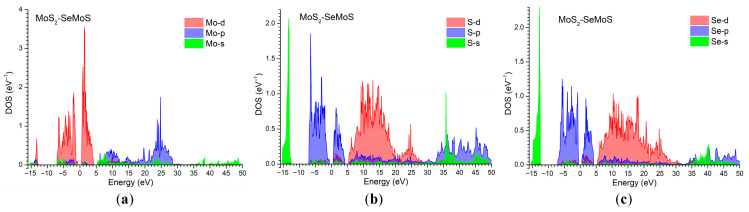
Partial DOS contributions of the MoS_2_/SeMoS heterostructure from the s, p, and d orbitals of the constituent atoms: (**a**) Mo; (**b**) S; (**c**) Se.

**Figure 6:**
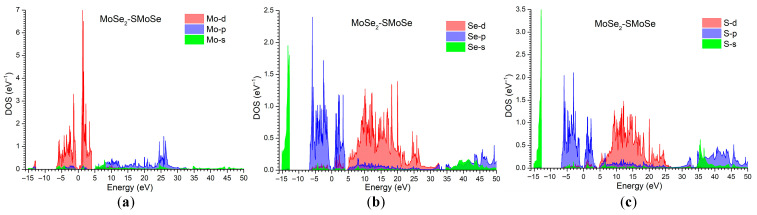
Partial DOS contributions of the MoSe_2_/SMoSe heterostructure from the s, p, and d orbitals of the constituent atoms: (**a**) Mo; (**b**) Se; (**c**) S.

Typically, to investigate the chemical bonding that underlies the structural ordering of nanosystems, the electronic density of states (DOS) associated with their constituent chemical elements is examined. For the considered vdW nano-heterostructures MoS_2_/SeMoS and MoSe_2_/SMoSe, the partial DOS contributions from the s, p, and d orbitals of Mo are shown in Figure 5 and Figure 6, respectively.

The analysis of the partial density of states (PDOS) for both Janus heterostructures—MoS_2_/SeMoS and MoSe_2_/SMoSe—reveals a pronounced orbital selectivity that determines the formation of the VB and CB edges. In both systems, the Mo d-orbitals play the dominant role, contributing to the top of the VB and the bottom of the CB, thereby confirming the characteristic d-type band edges typical of transition-metal dichalcogenides.

In the MoS_2_/SeMoS heterostructure, the p-states of S and Se predominantly occupy the upper part of the VB and strongly hybridize with Mo-d orbitals, forming the key bonding and antibonding states that govern the electronic structure near the bandgap. By contrast, the s-states of these chalcogens lie deep in the valence region (~−14…−15 eV) and do not participate in low-energy transitions. The presence of different chalcogen species on opposite sides of the Mo layer leads to a shift in the Se-p states relative to the S-p states, enhancing the intrinsic dipole moment and resulting in a noticeable asymmetry in the DOS distribution. This behavior reflects a significant electronic reconstruction induced by the Janus configuration and accounts for the high sensitivity of MoS_2_/SeMoS to external electric fields.

For MoSe_2_/SMoSe, the electronic states near the band edges are likewise governed by Mo-d orbitals; however, the distribution of the chalcogen p-states exhibits more pronounced asymmetry. Both S-p and Se-p states contribute to the formation of the VB, yet the Se-p orbitals display broader and more intense features than the S-p states, owing to the higher polarizability of selenium. This enhances the intrinsic dipole moment and leads to an even more substantial redistribution of the electronic density compared with MoS_2_–SeMoS. Such orbital asymmetry renders the MoSe_2_/SMoSe heterostructure particularly sensitive to external electric fields, as supported by its PDOS characteristics and consistent with general trends in Janus-type TMD materials.

Overall, the comparison of the PDOS of the two Janus heterostructures shows that the presence of different chalcogen layers causes a substantial rearrangement of the electronic states. Replacing sulfur with selenium in the upper TMD layer increases the dipole moment, enhances the Se-p contribution, and leads to stronger polarization of the electronic density. These features underscore the key role of Janus asymmetry in governing the sensitivity of such heterostructures to external electric fields and highlight their potential advantages for tuning electronic and optical properties.

**Figure 7:**
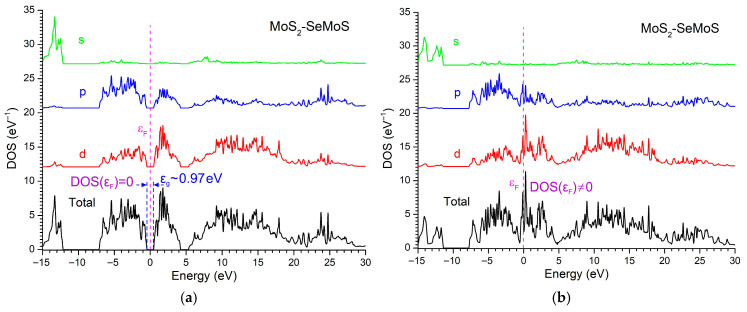
DOS of the MoS_2_/SeMoSnanoheterostructure in the absence of an external voltage (**a**) and under an applied voltage of 75 V (**b**). For clarity, the curves are shifted along the DOS axis.

The total and orbital-resolved DOS of the MoS_2_–SeMoS nanostructure demonstrate a clear semiconducting behavior at zero external field, with a bandgap of approximately 0.97 eV and DOS($\varepsilon_{F}$) = 0 (Figure 7a). The valence band is dominated by p-states of S and Se, while the conduction band originates mainly from Mo-d orbitals. In contrast, when an external field of 75 V is applied, the bandgap collapses and DOS($\varepsilon_{F}$) becomes finite, indicating a semiconductor-to-metal transition (Figure 7b). The external field significantly enhances the intrinsic Janus dipole, induces a pronounced redistribution of electronic states near $\varepsilon_{F}$, and leads to the emergence of Mo-d contributions at the Fermi level. This strong orbital reconstruction reflects the field-driven metallization characteristic of polar Janus TMD heterostructures.

**Figure 8:**
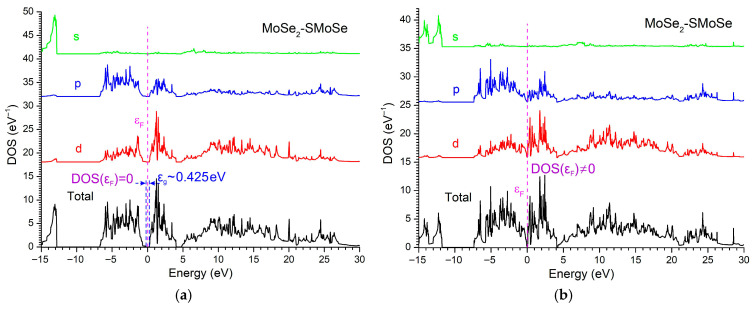
DOS of the MoSe_2_/SMoSenanoheterostructure in the absence of an external voltage (**a**) and under an applied voltage of 95 V (**b**). For clarity, the curves are shifted along the DOS axis.

The DOS of the MoSe_2_/SMoSe Janus heterostructure reveals semiconducting behavior at zero external bias, with DOS($\varepsilon_{F}$) = 0 and a bandgap of approximately 0.425 eV (Figure 8a). The VB maximum is dominated by the chalcogen p-states, while the CB minimum originates mainly from Mo-d orbitals. Under an external electric field of 95 V, the bandgap collapses and DOS($\varepsilon_{F}$) becomes finite, indicating a field-induced semiconductor-to-metal transition (Figure 8b). The applied electric field enhances the intrinsic Janus dipole and produces a substantial Stark-driven shift in the band edges, leading to a strong redistribution of the electronic density and the appearance of Mo-d states at the Fermi level. This metallization highlights the high tunability of MoSe_2_/SMoSe under external bias and underscores the distinct electric-field response of Janus TMD heterostructures.

**Figure 9:**
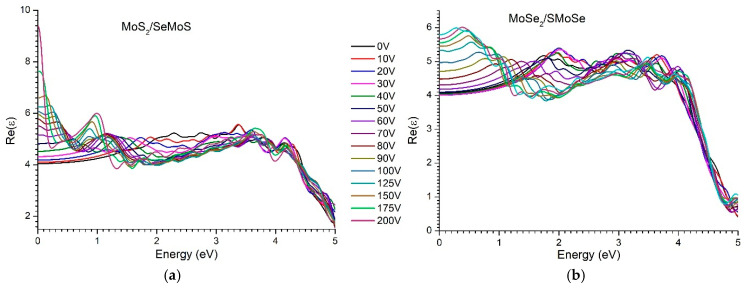
Evolution of the real part of the complex dielectric permittivity vdW of nanoheterostructures with increasing voltage from 0 to 200 V: (**a**) MoS_2_/SeMoSe; (**b**) MoSe_2_/SMoSe.

**Figure 10:**
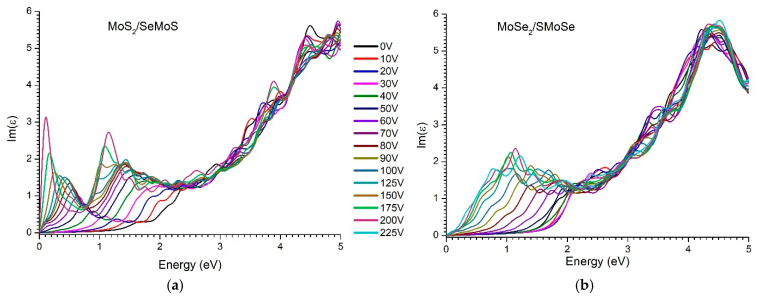
Evolution of the imaginary part of the complex dielectric permittivity vdW of nanoheterostructures with increasing voltage from 0 to 225 V: (**a**) MoS_2_/SeMoSe; (**b**) MoSe_2_/SMoSe.

Figure 9 and Figure 10 present the calculated real and imaginary parts of the complex dielectric function for the MoS_2_/SeMoS and MoSe_2_/SMoSenanoheterostructures, respectively, as a function of photon energy at different applied voltages (0–200 V). The real and imaginary components of the dielectric function were evaluated using Equations (1)–(5).

As is well known, the real part of the dielectric function, Re(ε), describes the ability of a material to polarize under an external electric field. It determines the refractive index and significantly influences the surface reflectivity (see Equation (4)). The values of the static dielectric permittivity ε_0_ for the MoS_2_/SeMoS and MoSe_2_/SMoSe nanostructures are 4.05 and 4.065, respectively. It should be noted that the static dielectric permittivity here refers to the value of ε(ω) at zero frequency, i.e., in the range between the phonon response and interband transitions.

As depicted in Figure 9a, with an increase in the bias voltage, the value of $\varepsilon_{g}$ also increases, which is consistent with expectations. According to the well-known Penn model [60], the static dielectric permittivity is inversely proportional to the bandgap width $\left(\varepsilon \propto \frac{1}{\varepsilon_{g}}\right)$, which can be described by the approximate expression:

(6) $$\varepsilon \approx 1+\left(\frac{\hbar \omega_{p}}{\varepsilon_{g}}\right),$$

where $\omega_{p}$ is the plasma frequency. Since the bandgap width decreases with an increase in the external electric field (see Figure 4), the value of $\varepsilon_{g}$ correspondingly increases according to Penn’s model, which is fully consistent with the obtained calculation results.

It is well known that the static dielectric permittivity characterizes the ability of a material to polarize in a constant electric field. Under the influence of an external electric field, the static dielectric permittivity $\varepsilon_{s}$ of the MoS_2_/SeMoS and MoSe_2_/SMoSe vdW nanoheterostructures increases significantly. In the absence of an external field, $\varepsilon_{s}$ is 4.049 and 4.065, respectively, whereas under an applied voltage of 60 V, these values increase to 4.819 and 4.18. Consequently, the response of Re(ε) to an external electric field is more pronounced in the MoS_2_/SeMoS nanoheterostructure than in the MoSe_2_–SMoSe system (Figure 9). The increase in $\varepsilon_{s}$ is governed by the redistribution of charge between the layers under the applied electric field, which enhances the overall polarization of the system.

As the applied voltage increases, Re(ε) exhibits a systematic shift in individual peaks toward lower photon energies. For example, in the MoS_2_–SeMoS structure, the Re(ε) peak located at ~2.1 eV at zero voltage is progressively shifted to ~1.9 eV (10 V), ~1.75 eV (20 V), …, and ~1.2 eV (60 V), respectively. This behavior indicates a reorganization of the electronic cloud and a change in the polarizability of the atomic layers under the influence of the external electric field.

Note that dielectric permittivity is related to the polarizability of an atom according to the well-known Clausius-Mosotti formula [61]:

(7) $$\frac{\varepsilon -1}{\varepsilon +2}=\frac{1}{3\varepsilon_{0}}\sum N_{j}a_{j},$$

where $N_{j}$ is the number of atoms per unit volume with polarizability $a_{j}$. (Traditionally, the polarizability of an atom is denoted by α, as in our case α denotes the absorption coefficient, we replaced it with the letter a). Usually, in the optical frequency range, the contribution to dielectric permittivity is determined exclusively by electronic polarizability. In this case, electronic polarization is strongly governed by the width of the bandgap, since it determines the energy required to excite electrons from the valence band to the conduction band. The behavior of the functions Re(ε) and Im(ε) in the optical range reflects the character of the electronic polarizability of atoms, which is caused by the displacement of the electron shell relative to the atomic nucleus. However, with the increasing influence of an external electric field in nanoheterostructures, the ionic polarizability begins to appear, which is connected with the displacement of charged ions relative to each other. Thus, the observed changes in the complex dielectric permittivity function of 2D vdW nanoheterostructures under the action of an external electric field can be explained by the increasing role of ionic polarizabilityand its growing contribution to the overall polarization response of the system.

In contrast to the MoS_2_/SeMoS structure, the MoSe_2_/SMoSenanoheterostructure demonstrates different behavior of the real part of the dielectric function Re(ε) when the external voltage changes. In the absence of an electric field, the main peak of Re(ε) is located in the region around 1.9 eV. When the voltage is increased to 30 V, the peak shifts to the high-energy region, reaching ~2.1 eV. However, with a further increase in voltage (starting from 40 V), the opposite shift in the peak to the low-energy region is observed—for example, at 50 V, the peak position is ~1.83 eV, and at 60 V, it is ~1.55 eV. This nonlinear shift in the Re(ε) peak is associated with the peculiarities of the evolution of the bandgap width under the action of an external electric field (see Figure 4b). In particular, first, the bandgap widens (up to ~20 V), and then it is seen narrowing at higher voltages, which directly affects the position and intensity of the peaks of the dielectric function.

As can be seen, the intensity of the imaginary part of the dielectric function, Im(ε), decreases at photon energies above ~4.5 eV (Figure 10b). We attribute this behavior to the reduced number of available interband transitions in this spectral region. The dominant optical transitions in MoSe_2_/SMoSe are concentrated near the fundamental absorption edge and within the valence and conduction bands located below ~4–4.5 eV. At higher energies, the contributions from the most densely populated electronic states are exhausted, and further transitions require promoting electrons to much higher-energy split bands, where the density of states is significantly lower. This results in a reduced probability of interband transitions and, consequently, a decrease in Im(ε). A similar attenuation of the high-energy spectral response is observed in other TMD and Janus structures, where the reduction in spectral intensity arises from the limited availability of transitions between distant electronic bands. Thus, the behavior of Im(ε) above ~4.5 eV reflects fundamental features of the electronic structure of the investigated heterostructure.

As can be seen from Figure 9 and Figure 10, the decrease in the real part Re(ε) occurs near the maximum of the imaginary part Im(ε). Note that this behavior of Re(ε) and Im(ε) from energies is regular and is a particular example of the dispersion relation of Kramers-Kroning [62]. It is also noticeable that as the value of the external electric field increases, the amplitude of the main peak Im(ε) decreases and shifts toward the low-energy region. For example, the peak of the MoS_2_/SeMoS structure at 1.74 eV shifts to 1.5 eV (at 10 V), 1.27 eV (at 20 V), 1.1 eV (at 30 V), …, 0.3 eV (at 150 V), i.e., the intensity of low-energy absorption gradually increases, and at the highest voltage values, an almost “metallic” low-energy—response occurs (approaching quasi-Drude behavior).

As can be seen, in the high-energy region of the spectrum (around 3 eV and above), the shape of the complex dielectric function remains practically unchanged with an increase in the external electric field (Figure 9 and Figure 10). This behavior can be explained by the fact that long-range interband transitions (occurring between deep energy levels) are less sensitive to the influence of a vertical electric field, because they involve more localized states which are weakly dependent on external polarization.

**Figure 11:**
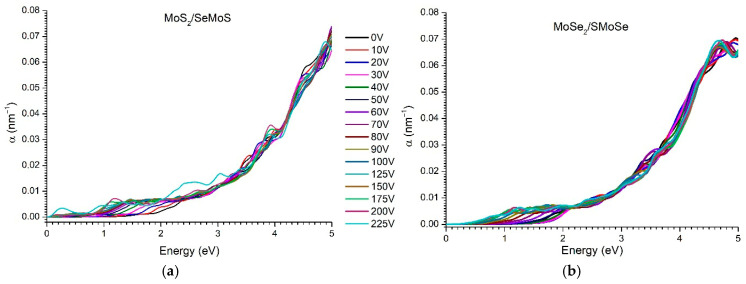
Evolution of the vdW absorption coefficient of nanoheterostructures with increasing voltage from 0 to 225 V: (**a**) MoS_2_/SeMoSe; (**b**) MoSe_2_/SMoSe.

**Figure 12:**
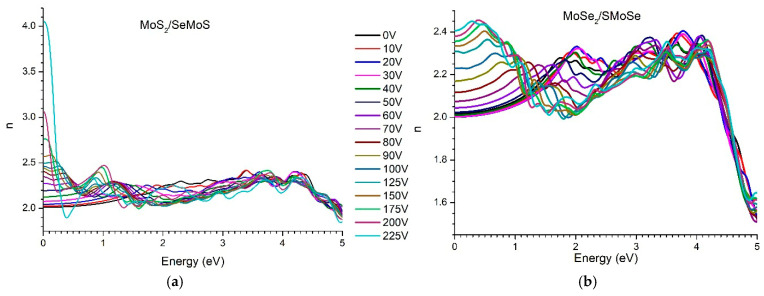
Evolution of the refractive index vdW of nanoheterostructures with increasing voltage from 0 to 225 V: (**a**) MoS_2_/SeMoSe; (**b**) MoSe_2_/SMoSe.

According to Equation (3), which follows from the Kramers-Kroning dispersion relation taking into account the properties of a nonmagnetic medium, the extinction coefficient k and optical absorption coefficient α significantly depend on the imaginary part of the dielectric permittivity Im(ε). Therefore, the main peaks of these functions usually occur at the same photon energy values, which reflects a direct relationship between light absorption and interband electron transitions. The results of calculating the absorption coefficient and refractive index of the considered vdW nanoheterostructures are shown in Figure 11 and Figure 12.

The optical absorption in the studied nanoheterostructures is determined by interband electron transitions and is described by the expression [63]:

(8) Imε,V∞∑c,υ,kMcυk2δEck−Eυk−ℏω,

where c, υ are indices of the conduction band and valence band, respectively, k is the wave vector (point in the Brillouin zone), $M_{c\upsilon }\left(k\right)$ is the matrix element of the dipole transition characterizing the probability of electron transition between bands, $E_{c}\left(k\right)$, $E_{\upsilon }\left(k\right)$ are energies of electrons in the conduction and valence bands, respectively, $\delta \left(x\right)$ is a delta function ensuring the conservation of energy during the transition.

Thus, the intensity of optical absorption is determined by the magnitude of the dipole-transition matrix elements and by the availability of states for transitions between the valence and conduction bands, both of which are sensitive to the bandgap width and the external electric field. As the bandgap width ε_g_(*V*) decreases, the minimum photon energy satisfying the δ-condition also decreases. Accordingly, the observed red shift in the absorption edge correlates directly with the reduction in ε_g_(*V*). The appearance of absorption peaks at low photon energies is associated with the emergence of increasingly “light”, i.e., low-energy, direct transitions—primarily K–K transitions, which correspond to the minimum bandgap values shown in Figure 4a.

In contrast to the MoS_2_/SeMoS nanoheterostructure, no low-energy peaks are observed in the absorption spectrum of the MoSe_2_/SMoSe vdW nanoheterostructure as the external voltage is increased (Figure 11b). The main spectral features remain concentrated in the region around 1 eV. This behavior is attributed to the absence of direct interband transitions in the MoSe_2_/SMoSe structure under increasing voltage (see Figure 4b). It is well established that direct transitions in k-space contribute most significantly to the intensity of optical absorption, whereas indirect (“oblique”) transitions of the K–Q or Γ–Q type, which require phonon participation, are considerably weaker.

The behavior of the refractive index of the studied nanoheterostructures follows the same trend as the real part of the complex dielectric permittivity (Figure 12). The refractive index increases significantly with increasing external voltage in the infrared range. For the MoS_2_/SeMoS structure, the refractive index changes from 2 to 4.05 as the voltage is varied from 0 to 225 V (Figure 12a). In contrast, the refractive index of the MoSe_2_/SMoSe structure is substantially lower and varies only between 2 and 2.4. This indicates that this structure responds much more weakly to the influence of the external electric field in comparison with the former (Figure 12b).

## 5. Conclusions

The investigation has established the dependence of the electronic and optical properties of the Janus nanoheterostructures MoS_2_/SeMoS and MoSe_2_/SMoSe on the external electric field. It has been shown that the geometric features and asymmetric atomic distribution within the layers create an intrinsic dipole, which enhances the sensitivity of these structures to the applied field.

For the MoS_2_/SeMoS structure, a nearly linear decrease in the bandgap width with increasing voltage has been observed, culminating in a transition to the metallic state at approximately 75 V. For MoSe_2_/SMoSe, a nonlinear evolution of the bandgap width has been revealed, with a maximum appearing at around 20 V, followed by a gradual narrowing leading to full closure at approximately 95 V. Calculations of the complex dielectric permittivity demonstrate that the external electric field results in an increase in the static dielectric permittivity, an enhancement of low-energy absorption, and a shift in spectral peaks toward lower energies. These effects are explained by charge redistribution and an increased contribution of ionic polarizability to the overall polarization response of the system.

Thus, the ability to control the electronic and optical characteristics of Janus nanoheterostructures using an external electric field has been demonstrated. The results confirm the high sensitivity of these systems and their potential for the development of tunable optoelectronic and photovoltaic devices based on electric-field-induced band-structure engineering.

## Data Availability

The original contributions presented in this study are included in the article. Further inquiries can be directed to the corresponding author.

## References

[1] Rafique H., Abbas G., Mendes M.J., Barquinha P., Martins R., Fortunato E., Águas H., Jana S. (2025). Recent Advancements and Perspectives of Low-Dimensional Halide Perovskites for Visual Perception and Optoelectronic Applications. Nano Micro Lett..

[2] Campaioli F., Gherardini S., Quach J.Q., Polini M., Andolina G.M. (2024). Colloquium: Quantum batteries. Rev. Mod. Phys..

[3] Myers N.M., Abah O., Deffner S. (2022). Quantum thermodynamic devices: From theoretical proposals to experimental reality. AVS Quantum Sci..

[4] Breczko J., Wysocka-Żołopa M., Grądzka E., Winkler K. (2024). Zero—Dimensional carbon nanomaterials for electrochemical energy storage. Chem Electro Chem.

[5] Kurniawan D., Xia Z., Dai L., Ostrikov K., Chiang W.-H. (2024). Zero-dimensional nano-carbons: Synthesis, properties, and applications. Appl. Phys. Rev..

[6] Sergeyev D., Duisenova A. (2022). Electron transport in core-shell type fullerene nanojunction. Adv. Nano Res..

[7] Ding M., Guo Z., Zhou L., Fang X., Zhang L., Zeng L., Xie L., Zhao H. (2018). One-Dimensional Zinc Oxide Nanomaterials for Application in High-Performance Advanced Optoelectronic Devices. Crystals.

[8] Zhou A.F., Feng P.X. (2024). One-Dimensional and Two-Dimensional Nanomaterials for Sensor Applications. Crystals.

[9] Sergeyev D. (2021). One-dimensional Schottky nanodiode based on telescoping polyprismanes. Adv. Nano Res..

[10] Maestre C., Li Y.D., Garnier V., Steyer P., Roux S., Plaud A., Loiseau A., Barjon J., Ren L., Robert C. (2022). From the synthesis of hBN crystals to their use as nanosheets in van der Waals heterostructures. 2D Mater..

[11] Hossain M., Zhao Z., Wen W., Wang X., Wu J., Xie L. (2017). Recent Advances in Two-Dimensional Materials with Charge Density Waves: Synthesis, Characterization and Applications. Crystals.

[12] Kim Y., Kim J. (2021). Near-field optical imaging and spectroscopy of 2D-TMDs. Nanophotonics.

[13] Ding D.D., Qu Z.Z., Han X.Y., Han C.R., Zhuang Q., Yu X.L., Niu R.R., Wang Z.Y., Li Z.X., Gan Z.Z. (2022). Multivalley Superconductivity in Monolayer Transition Metal Dichalcogenides. Nano Lett..

[14] Heil C., Ponce S., Lambert H., Schlipf M., Margine E.R., Giustino F. (2017). Origin of Superconductivity and Latent Charge Density Wave in NbS2. Phys. Rev. Lett..

[15] Tsuppayakorn-aek P., Pluengphon P., Phansuke P., Inceesungvorn B., Busayaporn W., Kaewtubtim P., Bovornratanaraks T. (2021). Effect of substitution on the superconducting phase of transition metal dichalcogenideNb(SexS1-x)2 van der Waals layered structure. Sci. Rep..

[16] Steinberg H., Simon S., Aprili M., Quay C.H.L. (2025). Transition Metal Dichalcogenide Superconductor Tunneling Devices: A Review. J. Supercond. Nov. Magn..

[17] Sergeyev D.M. (2012). About Tunneling of Pairs of the Cooper Pairs through the Josephson Junctions in Exotic Superconductors. Russ. Phys. J..

[18] Fiore S., Klinkert C., Ducry F., Backman J., Luisier M. (2022). Influence of the hBN Dielectric Layers on the Quantum Transport Properties of MoS2 Transistors. Materials.

[19] Sergeyev D. (2020). Single electron transistor based on endohedral metallofullerenes Me@C60 (Me = Li, Na, K). J. Nano Electron. Phys..

[20] Chen S., An Y., Wang S., Liu H. (2025). A Review of Tunnel Field-Effect Transistors: Materials, Structures, and Applications. Micromachines.

[21] Qian Y., Li Z., Chen J., Wang S. (2023). Van der Waals Integration of Artificial Heterostructures and Heterolayers. NSO J..

[22] Sun K., Zhang R., Liu H., Zhao T. (2025). Designable Excitonic Effects in Van der Waals Artificial Crystals with Exponentially Growing Thickness. Nat. Commun..

[23] Sgouros A.P., Michos F.I., Sigalas M.M., Kalosakas G. (2024). Thermal Relaxation in Janus Transition Metal Dichalcogenide Bilayers. Materials.

[24] Zhao H., Lam J.C.K. (2025). Preparation, Properties, and Applications of 2D Janus Transition Metal Dichalcogenides. Crystals.

[25] Zhu Z., Ren K., Shu H., Cui Z., Huang Z., Yu J., Xu Y. (2021). First-Principles Study of Electronic and Optical Properties of Two-Dimensional WSSe/BSe van der Waals Heterostructure with High Solar-to-Hydrogen Efficiency. Catalysts.

[26] Zhang Y., Tang T.-T., Girit C., Hao Z., Martin M.C., Zettl A., Crommie M.F., Shen Y.R., Wang F. (2009). Direct Observation of a Widely Tunable Bandgap in Bilayer Graphene. Nature.

[27] Scharf B., Frank T., Gmitra M., Fabian J., Žutić I., Perebeinos V. (2016). Excitonic Stark Effect in Monolayers. Phys. Rev. B.

[28] Sergeyev D., Ashikov N., Zhanturina N. (2021). Electric Transport Properties of a Model Nanojunction Graphene–Fullerene C60–Graphene. Int. J. Nanosci..

[29] Cao Y., Fatemi V., Fang S., Watanabe K., Taniguchi T., Kaxiras E., Jarillo-Herrero P. (2018). Unconventional Superconductivity in Magic-Angle Graphene Superlattices. Nature.

[30] Zhang L., Zhang Z., Wu F., Wang D., Gogna R., Hou S., Watanabe K., Taniguchi T., Kulkarni K., Kuo T. (2020). Twist-angle dependence of moiré excitons in WS_2_/MoSe_2_ heterobilayers. Nat. Commun..

[31] Sergeyev D., Duisenova A., Solovjov A., Ismayilova N. (2023). Electron transport in a stressed moiré bigraphene structure. Results in Phys..

[32] Sawtarie N., Schrecengost J., Ananthanarayanan K., Manimaran N., Awate S.S., Dong C., Xu K., Wang Y., Robinson J.A., Giebink N.C. (2025). Permanent Dipole Moment in a Quantum-Confined Two-Dimensional Metal Revealed by Electric Double Layer Gating. Nano Lett..

[33] Van Dyck C., Bergren A.J. (2018). Large Built-In Fields Control the Electronic Properties of Nanoscale Molecular Devices with Dipolar Structures. Adv. Electron. Mater..

[34] Conley H.J., Wang B., Ziegler J.I., Haglund R.F., Pantelides S.T., Bolotin K.I. (2013). Bandgap Engineering of Strained Monolayer and Bilayer MoS_2_. Nano Lett..

[35] Sun C., Zhong J., Gan Z., Chen L., Liang C., Feng H., Sun Z., Jiang Z., Li W.-D. (2024). Nanoimprint-Induced Strain Engineering of Two-Dimensional Materials. Microsyst. Nanoeng..

[36] Sergeyev D. (2018). Computer simulation of electrical characteristics of a graphene cluster with Stone-Wales Defects. J. Nano Electron. Phys..

[37] Sergeyev D., Duisenova A., Shunkeyev K. (2024). Electronic and Optical Properties of One-Dimensional Van Der Waals Nanodevices Based on MoS2(n,n) and MoSe2(n,n) Nanotubes. Crystals.

[38] Sergeyev D., Shunkeyev K. (2025). Transport Properties of One-Dimensional van der Waals Heterostructures Based on Molybdenum Dichalcogenides. Crystals.

[39] Ang Y.S., Yang S.A., Zhang C., Ma Z., Ang L.K. (2017). Valleytronics in merging Dirac cones: All-electric-controlled valley filter, valve and universal reversible logic gate. Phys. Rev. B.

[40] Vitale S.A., Nezich D., Varghese J.O., Kim P., Gedik N., Jarillo-Herrero P., Xiao D., Rothschild M. (2018). Valleytronics: Opportunities, Challenges, and Paths Forward. Small.

[41] Hudson R.J., MacDonald T.S.C., Cole J.H., Schmidt T.W., Smith T.A., McCamey D.R. (2024). A framework for multiexcitonic logic. Nat. Rev. Chem..

[42] Lopriore E., Tagarelli F., Fitzgerald J.M., Gonzalez Marin J.F., Watanabe K., Taniguchi T., Malic E., Kis A. (2025). Enhancing interlayer exciton dynamics by coupling with monolithic cavities via the field-induced Stark effect. Nat. Nanotechnol..

[43] Butera V. (2024). Density functional theory methods applied to homogeneous and heterogeneous catalysis: A short review and a practical user guide. Phys. Chem. Chem. Phys..

[44] Oh Y., Song S., Bae J. (2024). A Review of Bandgap Engineering and Prediction in 2D Material Heterostructures: A DFT Perspective. Int. J. Mol. Sci..

[45] Zheng S., Li C., Wang C., Ma D., Wang B. (2023). The Combined Effects of an External Field and Novel Functional Groups on the Structural and Electronic Properties of TMDs/Ti3C2 Heterostructures: A First-Principles Study. Nanomaterials.

[46] Li W., Wang T., Dai X.Q., Wang X., Zhang H., Zhong J., Luo J., Huang W. (2017). Electric Field Modulation of the Band Structure *in MoS*_2_/*WS*_2_ van der Waals Heterostructure. Solid State Commun..

[47] Liu Q., Li L., Li Y., Gao Z., Chen Z., Lu J. (2012). Tuning Electronic Structure of Bilayer MoS_2_ by Vertical Electric Field: A First-Principles Investigation. J. Phys. Chem. C.

[48] Guo F., Wang J., Gong Y., Wu J., Guo Y., Chen Y., Wu S. (2017). Modulation of Electronic and Optical Anisotropy Properties of ML-GaS by Vertical Electric Field. Nanoscale Res. Lett..

[49] Ramasubramaniam A., Naveh D., Towe E. (2011). Tunable Band Gaps in Bilayer Transition-Metal Dichalcogenides. Phys. Rev. B.

[50] Patel S., Kaloni T.P., Singh N., Schwingenschlögl U. (2022). Electric Field- and Strain-Induced Band-Gap Engineering and Manipulation of the Rashba Spin Splitting in Janus van der Waals Heterostructures. Phys. Rev. B.

[51] Perdew J.P., Burke K., Ernzerhof M. (1996). Generalized gradient approximation made simple. Phys. Rev. Lett..

[52] Khanjani S., Mohammadi-Manesh E., Ahmadvand N. (2025). Impact of metal doping on the electrical and optical properties of AgI_2_ QDs: A DFT study. Mater. Sci. Semicond. Process..

[53] Sergeyev D., Zhanturina N., Aizharikov A., Popov A.I. (2021). Influence of productive Impurities (Cd, Na, O) on the Properties of the Cu2ZnSnS4 Absorber of Model Solar Cells. Latv. J. Phys. Tech. Sci..

[54] Grimme S., Antony J., Ehrlich S., Krieg H. (2010). A Consistent and Accurate Ab Initio Parametrization of Density Functional Dispersion Correction (DFT-D) for the 94 Elements H–Pu. J. Chem. Phys..

[55] Monkhorst H.J., Pack J.D. (1976). Special Points for Brillouin-Zone Integrations. Phys. Rev. B.

[56] AtomistixToolKit with Virtual NanoLab ATK-VNL 2015.1. https://www.synopsys.com/.

[57] Wang G., Chernikov A., Glazov M.M., Heinz T.F., Marie X., Amand T., Urbaszek B. (2018). Excitons and Polaritons in Two-Dimensional Transition Metal Dichalcogenides. Rev. Mod. Phys..

[58] Silva-Guillén J.Á., San-Jose P., Roldán R. (2016). Electronic Band Structure of Transition Metal Dichalcogenides from Ab Initio and Slater–Koster Tight-Binding Model. Appl. Sci..

[59] Sergeyev D., Kenges A., Shunkeyev K. (2025). Electronic and Optical Properties of WS_2_(6,6)@MoS_2_(14,14) and WS_2_(8,8)@MoS_2_(16,16) van der Waals Nanotubes under the Influence of an External Electric Field. Adv. Nano Res..

[60] Penn D.R. (1962). Wave-Number-Dependent Dielectric Function of Semiconductors. Phys. Rev..

[61] Eremin I.E., Neshchimenko V.V., Shcherban D.S., Fomin D.V. (2021). System modification of the equation Lorenz–Lorentz–Clausius–Mossotti. Optik.

[62] Rousseau E., Izard N., Bantignies J.L., Felbacq D. (2021). Comment on the paper “Improving Poor Man’s Kramers-Kronig analysis and Kramers-Kronig constrained variational analysis. Spectrochim. Acta Part AMol. Biomol. Spectrosc..

[63] Martin R.M. (2004). Electronic Structure: Basic Theory and Practical Methods.

